# Water filtration by burrowing sandprawns provides novel insights on endobenthic engineering and solutions for eutrophication

**DOI:** 10.1038/s41598-020-58677-1

**Published:** 2020-02-05

**Authors:** Olivia Venter, Deena Pillay, Kervin Prayag

**Affiliations:** 1 FitzPatrick Institute of African Ornithology, DST-NRF Centre of Excellence, University of Cape Town, Cape Town, 7701 South Africa; 2 Marine Research Institute, Department of Biological Sciences, University of Cape Town, Cape Town, 7701 South Africa; 3 Department of Biological Sciences, University of Cape Town, Cape Town, 7701 South Africa

**Keywords:** Urban ecology, Ecosystem services

## Abstract

Managing coastal ecosystems and preserving socio-ecological functioning require a comprehensive understanding of ecological services provided by resident organisms. Here, we provide novel information on water-filtration activities of endobenthic sandprawns (*Callichirus kraussi*), which are key ecosystem engineers in South African coasts. We demonstrate experimentally that benthic engineering by sandprawns reduces phytoplankton biomass by roughly 50%. Using long-term estuarine data, we demonstrate similar reductions in phytoplankton biomass (by roughly 70%) in sandprawn-dominated areas. Increased burrow wall chlorophyll-*a* relative to surface sediments that was evident in experiments suggests that pelagic filtration occurs through bi-directional water pumping and phytoplankton adsorption onto burrow walls. Our findings expand understanding of the ecological relevance of sandprawns and functionally similar organisms, the mechanisms by which they engineer ecosystems and their role in mediating coastal bentho-pelagic coupling. Our findings also highlight the potential for deposit-feeders to be used as nature-based solutions to counter coastal eutrophication.

## Introduction

Coastal ecosystems are some of the most productive and ecologically significant habitats on earth^1–3^. They are critical nursery and feeding grounds for resident and migrant species and subsidise offshore ecosystems through nutrient and trophic transfers^4,5^. Coastal ecosystems also supply valuable goods and services that support human livelihoods and local economies^2,6^. However, due to their proximity to human settlements and their aesthetic appeal, coastal systems rank amongst the most threatened and degraded ecosystems on earth, owing to multiple human stressors being applied simultaneously^6–10^. Nutrient enrichment is arguably the most pervasive driver of coastal deterioration in the 21^st^ century, resulting in notable impairments of ecosystem multi-functionality and declines in the quality of goods and services provided^11,12^. Government and management agencies therefore invest heavily in measures to counter socio-ecological losses arising from excessive nutrient inputs.

The harnessing of ecological functions and services provided by biological systems to overcome environmental degradation, variously referred to as ecological engineering, nature-based solutions, bioremediation and/or biomanipulation, is increasing in popularity as a sustainable, nature-based tool that can mitigate nutrient enrichment in coastal ecosystems^12–15^. In this regard, suspension-feeding organisms (e.g. clams and mussels) and coastal vegetation have dominated mitigation narratives, leading to significant investments in conservation, restoration and engineering programs that exploit their water-purification capabilities^12,16,17^. In contrast, burrowing deposit-feeding organisms have been overlooked, despite them being dominant components of sedimentary biotopes (numerically and ecologically)^18–20^, which are significant constituents of coastal ecosystems. In particular, there has been a limited appreciation that burrow complexes produced by deposit-feeders can potentially function as water-filtration systems, with burrow-builders providing the water-pumping function^18,21,22^ and burrow walls and adjacent sediments the particle trapping function^23–25^.

Benthic deposit-feeders spend significant portions of their activity budgets on bi-directional water exchanges between pelagic and benthic ecosystems *via* active pumping through complex, sometimes interconnected burrow systems^21,26^. Such irrigation oxygenates burrow waters and eliminates faecal material, anoxic/hypoxic waters and potentially toxic compounds (e.g. H_2_S)^18,21^. Through such exchanges, suspended particles, including phytoplankton and other organic matter, can theoretically be adsorbed onto burrow walls and adjacent sediments^18,22–25^ in a manner akin to particle trapping achieved by artificial sediment filters. Particle adsorption is likely to be facilitated by the addition of mucus to burrow linings (for stability), which increases adsorptive properties. Additionally, microbial biofilms that line burrow walls can increase adhesive particle trapping further through exopolymer exudation^18,22^. Prior research demonstrating that burrow walls are enriched with microalgae, even at aphotic sediment depths, lends credence to the idea that burrow walls may act as sites of passive adsorption^23,24^. Consumption of adsorbed organic particles by burrow-builders, microbes and other infauna likely maintains the functional integrity of burrows as biological filters.

As is the case with artificial purification systems, pumping rate and filter surface-area are likely key determinants of filtration effectiveness in ecosystems dominated by deposit-feeders and their burrow systems. On this basis, endobenthic axiid crustaceans (formerly part of the Thalassinidea) are likely to exhibit the strongest filtration effects of extant benthic burrowers for several reasons. Firstly, these organisms produce intricate, interlinked burrow systems that extend several meters deep into sediments (2–3 m in some cases)^27–29^, increasing the area of the sediment-water interface several-fold. Secondly, they spend a significant portion of their daily activity budgets on bi-directional water pumping^26,30^, given that endobenthic sediments are prone to becoming hypoxic and hypercapnic^18^. Lastly, they occur as dense assemblages (up to 400 individuals/m^2^) with spatial occupancy spanning several kilometres^18,22^. These features suggest that in the presence of dense assemblages of axiid crustaceans, three-dimensional burrow superstructures may function as elaborate below-ground coastal filtration systems.

**Figure 1:**
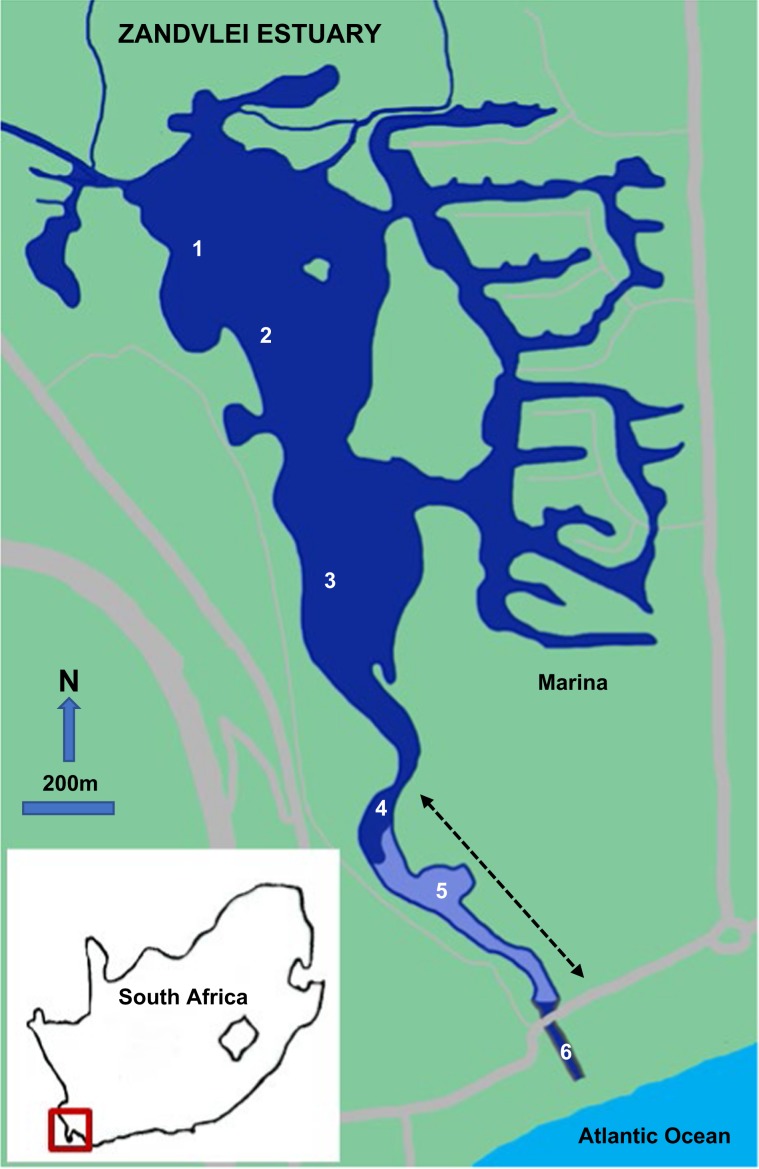
Map of the Zandvlei Estuary showing its geographical location with in South Africa (inset), position of sampling sites (1–6) and key features. Site 5 is located within a sandprawn-dominated biotope in the lower reaches, while Site 6 is located within a canalised region in which sandprawns are absent. Dashed arrow denotes the axial extent of the sandprawn biotope. Maps produced by Jessica Dawson and used with permission.

**Figure 2:**
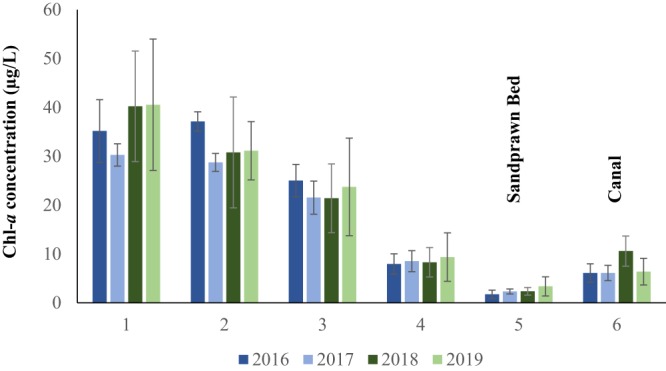
Inter-annual variability in *in situ* pelagic chl-*a* concentrations (means ± SD) among sites in the Zandvlei Estuary. Site 5 is located within a sandprawn-dominated biotope and Site 6 occurs in a canalised region in which sandprawns are absent. Water-column surface data are presented. SD is shown due to low data variance at Site 5.

While passive particle adsorption has previously been hypothesized as an explanation for greater levels of trophic resources along burrow walls^18,22,23^, effects on pelagic filtration have not been empirically tested, either in the context of axiid crustaceans or benthic deposit-feeders more broadly. This novel premise thus forms the scientific motivation for this paper, which tests the broad hypothesis that water quality would improve in the presence of the sandprawn *Callichirus kraussi*, which is a southern African burrowing axiid crustacean. More specifically, we hypothesised that if the burrow systems of *C. kraussi* function as biological filters, then phytoplankton biomass should decrease in the presence of this sandprawn. Our hypothesis was tested using a combination of *ex situ* mesocosm experiments and comparative *in situ* approaches in the Zandvlei Estuary Nature Reserve (Fig. 1), which was the focal system of this study. Our hypothesis was additionally driven by observations of water clarity generally being greater in biotopes where *C. kraussi* is abundant in the Zandvlei Estuary (See Fig. 2 in results for supporting data). *C. kraussi* is a dominant ecosystem engineer in coastal ecosystems locally (including highly eutrophic, urban systems), where it influences multiple resource flows that significantly determine compositional and structural attributes of biotic assemblages^24,31–34^. More broadly, our work was intended to expand understanding of the functional relevance of a globally distributed group of endobenthic organisms that are highly influential ecosystem engineers in coastal habitats^18,22^.

## Materials and Methods

### Study site

**Figure 3:**
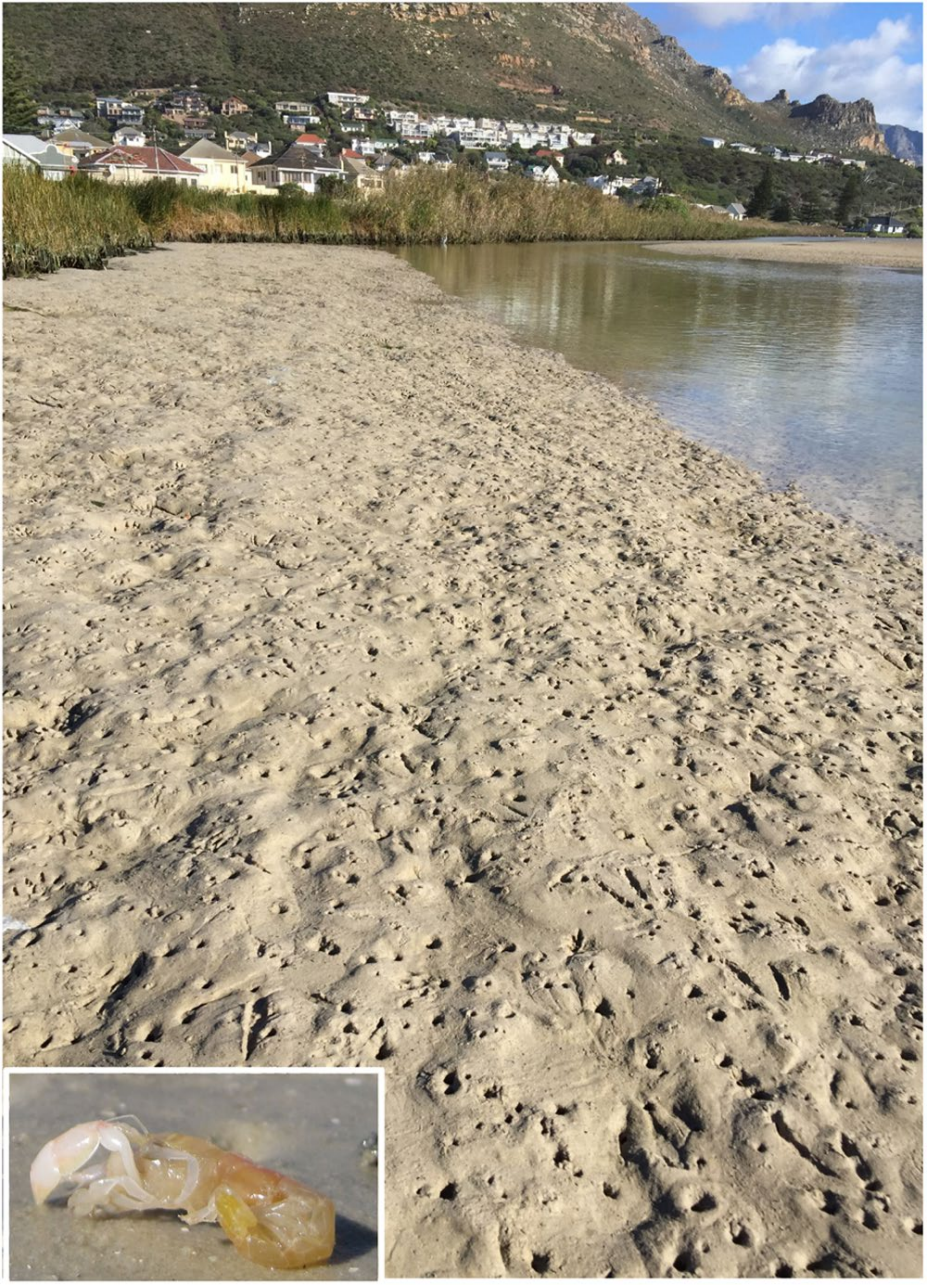
Irregular sediment topography created by dense *Callichirus kraussi* (inset) populations in the sandprawn-dominated biotope in the Zandvlei Estuary. Holes on the sediment surface are burrow openings. The main image was taken 3 days after mouth opening, during which drainage into the Atlantic Ocean exposed most of the benthic habitat. Inset courtesy of Jessica Dawson, used with permission.

The Zandvlei Estuary is a shallow urban system (mean depth = 1.4 m)^35^ located roughly 30 km south of Cape Town, South Africa (Fig. 1). The system has historically undergone significant anthropogenic modification, including the construction of a marina in the east, the canalisation of the lower reaches and artificial manipulation of the mouth to manage water levels^35^. The northern parts of the system are highly eutrophic^35^, due mainly to riverine inputs from surrounding urban areas, including large informal settlements with poor sanitation infrastructure. The estuary mouth is mechanically opened to the Atlantic Ocean approximately once per month for roughly a week during summer (low rainfall) and kept open during the winter (high rainfall) months. The lower reaches of the system is occupied by dense aggregations (176/m^2^–240/m^2^) of the sandprawn *Callichirus kraussi*, but this biotope accounts for approximately 4.9% of the total area of the system (Figs. 1 and 3).

### Experimental design

A two-week indoor mesocosm experiment was conducted at the aquarium facility at the University of Cape Town (UCT) to quantify the filtration function provided by sandprawns. Nine mesocosms (diameter = 27 cm, volume = 25 L) were filled with sediment (depth = 20 cm) and water (depth = 20 cm) from the Zandvlei Estuary. Sediment was collected from a sandprawn-dominated biotope in the lower reaches and sieved (2 mm mesh) before being added to each mesocosm. Estuarine water (salinity = 20‰, temperature = 17 °C) was collected approximately 200 m upstream of the sandprawn biotope. Individual mesocosms were aerated and left for one hour before initial (Day 0) water quality data were collected and sandprawns added to the designated mesocosms. To ensure uniformity across mesocosms and to maintain conditions to support sandprawn survival, conductivity, dissolved oxygen, pH and temperature were measured in each mesocosm on Day 0 and every 2 days thereafter, using a multi-probe water sampler (Lovibond® Water Testing SensoDirect 150 with PT1000). The experiment comprised three sandprawn treatments - controls lacking sandprawns, 50% natural density (6 sandprawns/mesocosm) and 100% natural density (11 sandprawns/mesocosm), with 3 replicates of each. The 100% density treatment corresponded to upper ranges of sandprawn densities (200 sandprawns/m^2^) reported in South African estuaries^24^.

Sandprawns were collected from the Zandvlei Estuary using standard stainless-steel prawn pumps (length = 75 cm, diameter = 5 cm). Only non-ovigerous sandprawns, approximately 4 cm to 8 cm in length (chela to telson), were selected. After extraction from the sediment, the sandprawns were placed in loose, moistened layers of newspaper and transported to the aquarium facility at UCT, where they were distributed amongst the mesocosms according to the designated treatments. Similar sizes of sandprawns were added to each mesocosm to prevent density effects being confounded by size variation. During the experiment, the number of burrow openings in each mesocosm was monitored to approximate the number of sandprawns per mesocosm. Past studies have shown that the number of burrow openings closely corresponds to sandprawn abundance^36–38^.

Prior to sandprawn additions to mesocosms (Day 0), and every 2 days thereafter until Day 11, 3 mL water samples were collected from each mesocosm to measure chlorophyll-*a* (chl-*a*) concentrations. Two water samples were taken from each mesocosm at depths of 6 cm (lower depth) and 12 cm (upper depth) above the sediment surface, using a syringe fitted with a fine tube. This was done to determine if potential sandprawn effects were localised and therefore depth-specific. Chl-*a* concentrations were determined fluorometrically (Turner Designs Trilogy). On days 1, 7 and 9, 40 mL water samples were collected from the lower and upper depth levels of each mesocosm to measure concentrations of phosphate (PO_4_^3−^), ammonium (NH_4_^+^), nitrate (NO_3_^−^) and nitrite (NO_2_^−^), using a multiparameter research photometer (Hanna Instruments HI 83203). On day 11, after final pelagic chl-*a* measurements were made, surface water samples (500 mL) were collected from mesocosms and filtered (Whatman GFF) to quantify total suspended sediment loads. This was followed by filters and sediment being dried (70 °C) overnight. The difference in mass of filters before and after filtration of mesocosm water was used to quantify suspended sediment loads. At the termination of the experiment (2 weeks), each mesocosm was drained completely (four 3 mm holes drilled at mesocosm bases) and 6 randomly interspersed surface sediment cores (diameter = 1.5 cm, depth = 1 cm) were collected from each of the 50% and 100% mesocosms. Three samples were collected from burrow mounds, and the remaining 3 from between mounds; these were pooled to generate a single mean value per mesocosm. Additional samples were collected from the burrow walls by sectioning the burrow to a depth of 1 cm and carefully scraping the sand away to expose the burrow wall^38^. Three different burrow lining samples were collected per mesocosm and averaged per mesocosm. Three surface sediment cores per mesocosms were also collected from controls. Sediment chl-*a* concentrations were determined fluorometrically (Turner Designs Trilogy) following extraction in 30 mL of 90% acetone and refrigeration for 46 hours. Chl-*a* concentrations were expressed relative to mass of sediment sample collected^38^. Sandprawns were returned to the Zandvlei Estuary at the termination of the experiment.

### Field comparisons

Long-term monitoring data (2016–2019) from the Zandvlei Estuary were used to determine (1) whether chl-*a* biomass differed between sites with and without sandprawns and (2) if trends recorded in mesocosm experiments were also evident *in situ*. For each year of monitoring, surface chl-*a* data (depth = 10–20 cm from surface) were collected at 6 sites (Fig. 1) along the axial length of the estuary using a factory-calibrated YSI 650MPI multiprobe fitted with a chl-*a* sensor. Five chl-*a* measurements were taken per site per year during the March–April period. In October 2018, surface water samples (10–20 cm from surface) for suspended sediment load determinations were collected at Site 5 (n = 5), which occurs within the sandprawn-dominated biotope and Site 4 (n = 5), which is a sandprawn-free site located upstream of the sandprawn biotope (Fig. 1). Suspended sediment loads were determined as outlined for the mesocosm experiment. Chl-*a* and suspended sediment data were collected during closed-mouth phases, with within-site replicates being collected at distances between 100 and 200 m.

### Data analysis

All data analyses were performed using the R data analysis platform^39^. Linear Models (LMs) were fitted to test effects of sandprawn density and day on conductivity, dissolved oxygen, pH and water temperature in experimental mesocosms using the *‘stats’* package^39–41^. Linear Mixed-Effects Models (LMMs) were fitted by restricted maximum likelihood (REML) estimation using the *‘lme4’* package^42^ to determine effects of sandprawn density, water depth, and sediment position (where relevant) on pelagic and benthic chl-*a* and nutrient concentrations. Where water depth did not explain significant variation in response variables, surface data were used in subsequent analyses. In cases where data were collected over multiple days, the days were considered a random effect, since concentrations were expected to change over time within each treatment. The significance of main effects in the LMs and LMMs was determined using the *‘car’* package^43^. In the cases where main effects significantly explained variation (p < 0.05), multiple comparisons of means (Tukey Contrasts) using the *‘emmeans’* package^44^ were applied to the LMs and LMMs to identify within-group and within-treatment differences. A posterior predictive simulation was used to evaluate model fits. The final LMM was evaluated using interquartile ranges as the summary statistic. The model was deemed to represent the data adequately if the posterior predictive p-value was greater than 0.78^42^, indicating that at least 78% of observed data fell within the simulated distribution^42^.

### Ethics statement

Experiments were conducted in accordance with guidelines of the University of Cape Town. Approval of the research was granted by the University of Cape Town, Science Faculty Animal Ethics Committee (approval number 2018/v10/DP).

## Results

Abiotic conditions were generally uniform among sandprawn treatments across sampling days (Table 1). Dissolved oxygen (F_8,36_ = 0.06, p = 1.000), pH (F_8,36_ = 0.22, p = 0.986) and temperature (F_8,36_ = 0.15, p = 0.996) did not vary across mesocosms for the study period. Significant variation in conductivity (F_8,36_ = 5.94, p < 0.0001) was detected, which was explained by sandprawn density (F_2,38_ = 11.23, p = 0.0001) and days (F_4,38_ = 9.86, p < 0.0001), but not their interaction (F_8,30_ = 0.18, p = 0.991). Conductivity generally increased over time and with increasing sandprawn density, but conductivity increases were minor, with mean levels ranging between 26.7 mS/cm ± 0.41 SE and 30.3 mS/cm ± 0.10 SE over time. Conductivity did not differ by more than 2 units between mesocosms with and without sandprawns over the duration of the study.

**Table 1:** Temporal variability in temperature, pH, dissolved oxygen and conductivity (means ± SE) among sandprawn density treatments (50% = 6 sandprawns/mesocosm; 100% = 11 sandprawns/mesocosm).

Day	Sandprawn treatment	Temperature (°C)	pH	Dissolved oxygen (mg/L)	Conductivity (mS/cm)
0	Control	17.4 ± 0.14	8.2 ± 0.00	9.4 ± 0.01	26.7 ± 0.41
	50%	17.4 ± 0.22	8.2 ± 0.01	9.4 ± 0.01	27.2 ± 0.13
	100%	17.2 ± 0.15	8.2 ± 0.00	9.2 ± 0.10	27.6 ± 0.30
2	Control	13.6 ± 0.05	8.3 ± 0.00	9.4 ± 0.03	27.4 ± 0.36
	50%	13.5 ± 0.05	8.3 ± 0.00	9.3 ± 0.03	29.0 ± 0.05
	100%	13.5 ± 0.08	8.2 ± 0.01	9.2 ± 0.03	29.3 ± 0.10
4	Control	13.2 ± 0.07	8.3 ± 0.00	10.2 ± 0.04	28.2 ± 0.42
	50%	13.0 ± 0.08	8.3 ± 0.00	10.2 ± 0.04	29.6 ± 0.07
	100%	13.0 ± 0.09	8.3 ± 0.01	10.2 ± 0.05	29.8 ± 0.13
6	Control	13.6 ± 0.04	8.3 ± 0.00	10.4 ± 0.01	28.1 ± 0.47
	50%	13.5 ± 0.06	8.4 ± 0.00	10.5 ± 0.01	29.7 ± 0.05
	100%	13.4 ± 0.08	8.3 ± 0.01	10.4 ± 0.04	30.0 ± 0.10
8	Control	13.8 ± 0.05	8.4 ± 0.00	9.7 ± 0.04	28.8 ± 0.47
	50%	13.8 ± 0.07	8.4 ± 0.00	9.6 ± 0.04	30 ± 0.06
	100%	13.7 ± 0.08	8.4 ± 0.00	9.8 ± 0.09	30.3 ± 0.10

Data from the 2-week mesocosm experiment are shown.

Water depth did not significantly explain variation in NO_2_^−^ (*χ*^2^ = 0.17, df = 1, p = 0.684), NH_4_^+^ (*χ*^2^ = 0.01, df = 1, p = 0.931), PO_4_^3−^ (*χ*^2^ = 0.001, df = 1, p = 0.988) and chl-*a* concentrations (*χ*2 = 0.01, df = 1, p = 0.928)in the mesocosms. NO_3_^−^ levels were below the 0.0 mg/L detection limit for all mesocosms. Sandprawn density significantly explained variation in mesocosm NH_4_^+^ concentrations (*χ*^2^ = 20.85, df = 2, p < 0.001), which were roughly 2.5 times greater in the 100% treatment relative to controls. Variation in PO_4_^3−^ (*χ*^2^ = 0.56, df = 2, p = 0.754) and NO_2_^−^ (*χ*^2^ = 2.28, df = 2, p = 0.320) concentrations were not explained by sandprawn density (Table 2).

**Table 2:** Temporal variability in nutrient concentrations (means ± SE) among sandprawn density treatments (50% = 6 sandprawns/mesocosm; 100% = 11 sandprawns/mesocosm).

	Sandprawn Treatment	Water column level	Concentration on Day 1 (mg/L)	Concentration on Day 7 (mg/L)	Concentration on Day 9 (mg/L)
NO_2_^−^	Control	Surface	0.04 ± 0.01	0.13 ± 0.04	0.26 ± 0.14
		Bottom	0.04 ± 0.01	0.13 ± 0.05	0.27 ± 0.14
	50%	Surface	0.05 ± 0.01	0.015 ± 0.03	0.23 ± 0.04
		Bottom	0.04 ± 0.01	0.015 ± 0.03	0.23 ± 0.04
	100%	Surface	0.06 ± 0.02	0.08 ± 0.005	0.11 ± 0.02
		Bottom	0.06 ± 0.02	0.07 ± 0.003	0.12 ± 0.01
PO_4_^3−^	Control	Surface	0.03 ± 0.02	0.85 ± 0.82	0.26 ± 0.23
		Bottom	0.03 ± 0.03	0.84 ± 0.81	0.07 ± 0.05
	50%	Surface	0.08 ± 0.01	0.57 ± 0.49	0.17 ± 0.10
		Bottom	0.09 ± 0.03	0.26 ± 0.09	0.27 ± 0.04
	100%	Surface	0.05 ± 0.04	0.25 ± 0.03	0.31 ± 0.13
		Bottom	0.04 ± 0.04	0.24 ± 0.18	0.26 ± 0.16
NH_4_^+^	Control	Surface	0.82 ± 0.07	0.96 ± 0.12	1.06 ± 0.09
		Bottom	0.76 ± 0.17	1.08 ± 0.11	0.82 ± 0.09
	50%	Surface	0.79 ± 0.19	1.45 ± 0.10	2.01 ± 0.04
		Bottom	0.66 ± 0.10	1.46 ± 0.06	1.98 ± 0.82
	100%	Surface	1.03 ± 0.49	1.72 ± 0.42	2.69 ± 0.82
		Bottom	0.62 ± 0.04	1.67 ± 0.41	3.30 ± 1.42

Data (surface and bottom) from the 2-week mesocosm experiment are shown.

**Figure 4:**
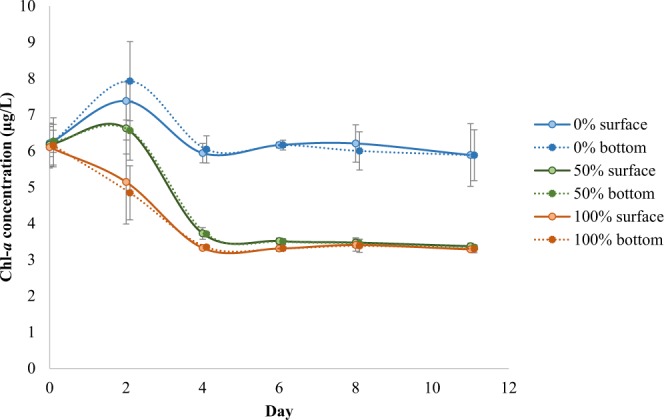
Temporal variability in pelagic chl-*a* concentrations (means ± SE) among sandprawn density treatments (0% = control; 50% = 6 sandprawns/mesocosm; 100% = 11 sandprawns/mesocosm). Surface and bottom data from the 2-week mesocosm experiment are shown.

Sandprawn density was a significant predictor of variability in water column chl-*a* concentrations in experimental mesocosms over the duration of the study (*χ*^2^ = 105.41, df = 2, p < 0.001; Fig. 4). At the termination of the experiment, pelagic chl-*a* concentrations were reduced by roughly 45% in the mesocosms containing sandprawns. In the 50% density, mean chl-*a* concentration was reduced by 45.1% from 6.2 ± 0.34 SE μg/L to 3.4 ± 0.05 SE μg/L. Similarly, reductions in mean chl-*a* levels by 45.9% from 6.1 ± 0.33 SE μg/L to 3.3 ± 0.06 SE μg/L were recorded at 100% sandprawn density. In the controls, pelagic chl-*a* concentration declined by 4.8% over the entire study period, with an initial concentration of 6.2 ± 0.21 SE μg/L and a final concentration of 5.9 ± 0.16 SE μg/L, (Fig. 4).

Patterns recorded in experimental mesocosms, in which sandprawn presence was associated with low chl-*a* concentrations, were mirrored by long-term field data. More specifically, over the 2016–2019 period, water column chl-*a* levels were consistently lowest within the sandprawn-dominated biotope (Site 5, Fig. 1), with mean levels ranging between 1.74 ± 0.38 SE μg/L and 3.36 ± 0.87 SE μg/L (Fig. 2). At the site immediately upstream of the sandprawn biotope (Site 4, Fig. 1), chl-*a* levels were greater and ranged between 7.94 ± 0.93 SE μg/L and 9.36 ± 2.21 SE μg/L. Similarly, at the canalised downstream site (Site 6, Fig. 1) where sandprawns were absent, mean chl-*a* levels were greater than in the sandprawn biotope, ranging between 6.09 ± 0.84 SE μg/L and 10.60 ± 1.38 SE μg/L. The northern-most sites sampled (Sites 1–3, Fig. 1) were highly eutrophic, with minimum and maximum mean chl-*a* concentrations of 21.40 ± 3.14 SE μg/L and 40.55 ± 6.01 SE μg/L being recorded (Fig. 2).

**Figure 5:**
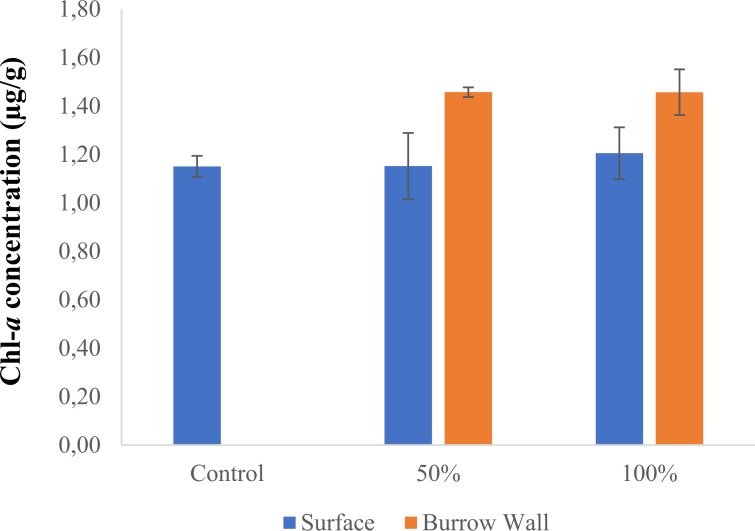
Variation in chl-*a* concentrations (means ± SE) between burrow walls and sediment surface samples between control and 50% (6 sandprawns/mesocosm) and 100% (11 sandprawns/mesocosm) sandprawn density treatments. Data from the 2-week mesocosm experiment are shown.

**Figure 6:**
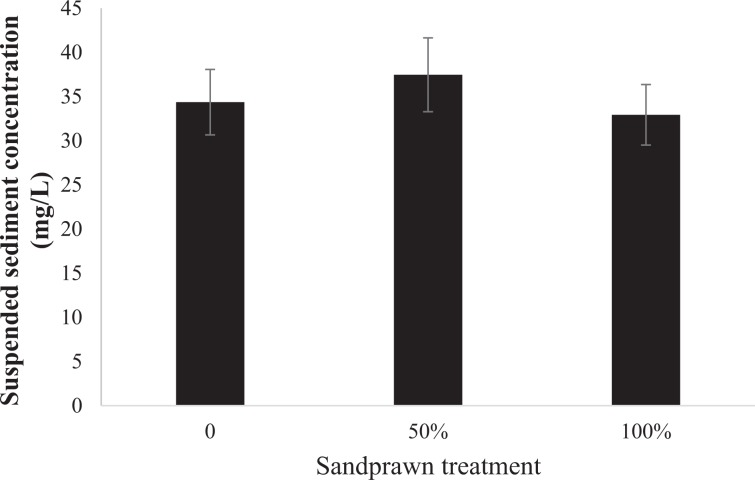
Differences in suspended sediment loads (means ± SE) among sandprawn density treatments (0% = control; 50% = 6 sandprawns/mesocosm; 100% = 11 sandprawns/mesocosm). Surface data from the 2-week mesocosm experiment are shown.

**Figure 7:**
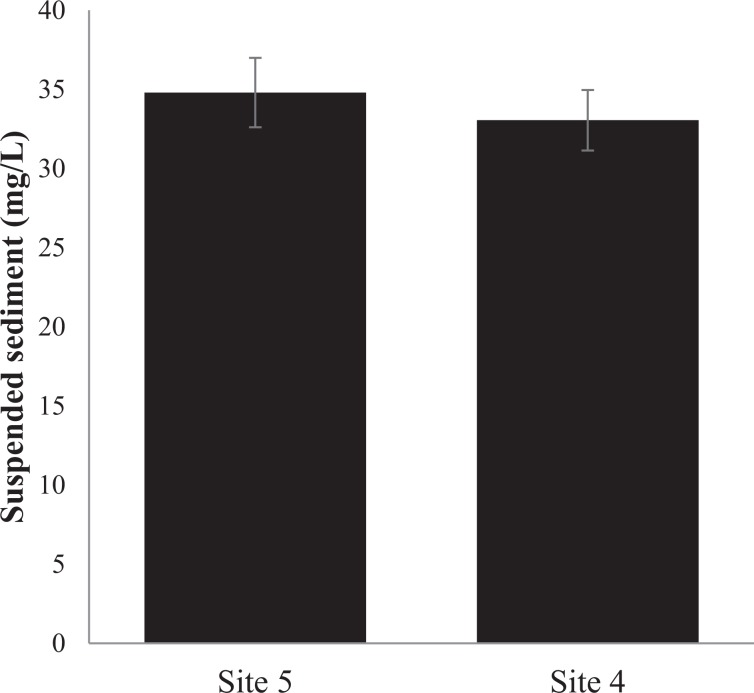
Variability in *in situ* pelagic suspended sediment loads (means ± SD) between Site 5, which is located within a sandprawn-dominated biotope and Site 4, which is located upstream of the sandprawn-dominated biotope. Surface data are presented.

Sediment chl-*a* biomass did not vary between the controls and 50% and 100% sandprawn density treatments (F_2,6_ = 0.29, p = 0.75; Fig. 5). However, in sandprawn treatments, position with the sediment did influence chl-*a* biomass, with burrow chl-*a* levels being between 13% (100% sandprawn treatment) and 20% (50% sandprawn treatment) greater than surface values (F_1,6_ = 3.76, p = 0.01; Fig. 5). Suspended sediment loads did not vary between controls and sandprawn treatments in the mesocosm experiment (F_1,6_ = 1.12, p = 0.38; Fig. 6) or between the sandprawn-dominated site (Site 4) or the upstream sandprawn free site (Site 5; F_1,8_ = 0.86, p = 0.37; Fig. 7).

## Discussion

Findings from our mesocosm experiment provide compelling evidence that sandprawn presence is linked causally to improvements in water quality. This is apparent through the near-50% reduction in phytoplankton biomass (chl-*a*) in overlying waters over the 11-day experimental period, with filtration effects manifesting after roughly 4 days. A decline in chl-*a* by approximately 70% in the sandprawn dominated biotope relative to the sandprawn-free site immediately upstream in the Zandvlei Estuary, also supports the notion of sandprawn presence being linked to improved water quality. Lastly, the increase in pelagic chl-*a* in the downstream canalised section of the estuary, where sandprawns are absent, adds further credence to the idea that improved water quality is associated with sandprawn presence. Overall, the broad agreement between findings of the mesocosm experiment and long-term *in situ* datasets strengthens confidence in ascribing a water-filtration function to sandprawns. Taken collectively, findings support our hypothesis that sandprawn presence would lead to improvements in water quality, by reducing phytoplankton biomass (measured as chl-*a*). At a broader level, our findings add to knowledge of processes that influence coastal water column productivity, given that physico-chemical processes, and filter feeders from a biotic perspective, have generally dominated perspectives in this field. Our findings however, suggest that engineering activities of sandprawns and similar organisms may be responsible for exerting a form of top-down regulation of coastal productivity.

Given that sandprawns are not filter-feeding organisms^45^, the pelagic filtration effects that we recorded are likely driven by phytoplankton adsorption onto burrow walls during bi-directional water pumping. Our data are supportive of this idea, since chl-*a* levels were on average 13% to 20% greater along burrow walls than surface sediments in experimental mesocosms containing sandprawns. It could be argued that greater burrow-wall chl-*a* levels that we recorded may be driven by phytoplankton adsorption onto wall during drainage of mesocosms at the end of the experiment. However, this is unlikely to be the sole explanation for the differentials that we recorded for several reasons. Firstly, controls were also drained in the exact manner as described for the +sandprawn treatments, but surface chl-a levels in this treatment were similar to values in the +sandprawn treatments, despite controls having roughly 50% greater pelagic chl-*a* concentrations. Secondly, if it is accepted that drainage may have been responsible for higher burrow-wall chl-*a* levels through adhesion and trapping, then sandprawn effects cannot be ignored, given that for 11 days over which pelagic chl-*a* data were collected, they would have been pumping water in and out of burrows, effectively facilitating phytoplankton trapping over this period. If burrows did not have any phytoplankton trapping effect, then it would have been unlikely for the declining water column chl-*a* effects that we recorded to manifest. This argument is supported by the lack of differences in suspended sediment loads between sandprawn presence and absence in experimental or *in situ* data, which suggests that the declines in pelagic chl-*a* levels that we recorded in the presence of sandprawns were unrelated to increased turbidity associated with sandprawn bioturbation.

Our findings regarding burrow chl-*a* concentrations are similar to those of Branch and Pringle^24^, who reported greater *in situ* chl-*a* concentrations closer to sandprawn (*Callichirus kraussi*) burrow walls and with increasing sediment depth. Similarly, studies on *Pestarella tyrrhena* have reported greater chl-*a* levels in burrow walls than surface sediments^23^. Our phytoplankton adsorption hypothesis is additionally supported by prior research in which direct observations of sandprawn (*C. kraussi*) behaviour within narrow-walled benthic chambers indicated an absence of filter-feeding activity^26^. Moreover, sandprawn burrows are irregular in shape^46^, and not ‘U’ or ‘Y’ shaped, which is typical of filter-feeding crustaceans^47^. Collectively, empirical data from our mesocosm experiment allied with prior research suggests that burrow-wall adsorption of phytoplankton cells is the probable driver of sandprawn-induced declines in phytoplankton biomass.

Our finding from the mesocosm experiment that NH_4_^+^ concentrations in overlying waters increased with sandprawn density is not entirely surprising, given that NH_4_^+^ is the most available of the dissolved nitrogen species for exchange across the sediment-water interface^18^. Increased fluxes of NH_4_^+^ in the presence of burrowing organisms are not uncommon and is thought to be driven by burrower excretion, greater diffusive area (burrows) and nitrogen mineralisation either through increased bacterial activity or organic matter content within burrows^25,48–53^. Increasing NH_4_^+^ fluxes have on occasion been linked to increases in primary abundance and/or productivity^52^. In our experiment however, despite the increase in pelagic NH_4_^+^ concentrations with increasing sandprawn density, there was no obvious effect on net pelagic filtration by sandprawns, since phytoplankton biomass declined to a steady state after 4 days in the presence of sandprawns, whereas NH_4_^+^ levels increased with sandprawn density over time. This would suggest that sandprawn filtration is robust against any potential positive effect of NH_4_^+^ fluxes on phytoplankton biomass.

At a broad level, the pelagic filtration that we have identified for sandprawns is novel, sheds new light on the functional significance of these and similar deposit-feeders in coastal ecosystems, and expands understanding of mechanisms by which they engineer marine ecosystems. To date, research in these areas has been dominated by bentho-centric ideas, in which ecosystem engineering activities (biogeochemical cycling, geotechnical characteristics, community regulation) in sedimentary biotopes have been emphasised^18–20,22,54^. Current paradigms are therefore built around the idea that while resource modulation and emergent engineering effects of deposit-feeders are large, particularly in the case of endobenthic species, they are generally restricted to the benthos^18–20,22,54^. Our findings show however, that engineering effects may not necessarily be confined to the benthos. While engineering activities are primarily benthic, by virtue of burrow construction within sediments, secondary engineering effects can manifest in pelagic ecosystems due to phytoplankton adsorption onto burrow walls. This secondary engineering function (pelagic filtration) has not previously been ascribed to deposit-feeding organisms and has traditionally been considered the domain of benthic filter-feeders. On a similar note, pelagic filtration by deposit-feeders has not featured in existing paradigms on bentho-pelagic coupling in coastal ecosystems, with narratives emphasising coupling by filter-feeders through phytoplankton consumption and by endobenthic engineers through material fluxes (mainly nutrients) into the water column^55^. Our findings however, demonstrate that endobenthic burrowers can couple benthic and pelagic ecosystems through pelagic filtration, and highlight the need for further research on this phenomenon to understand its dimensions more broadly. This assumes great significance when viewed in the context of existing knowledge gaps regarding bentho-pelagic coupling processes, particularly those mediated by organisms^55^. Also relevant are concerns emanating from the latter about limitations in predictive capabilities regarding coastal responses to global change^55^.

At its broadest level, our findings highlight the potential for ecosystem engineering activities of sandprawns and functionally similar deposit-feeders to increase coastal resilience against nutrient enrichment by limiting phytoplankton biomass. To date, endobenthic deposit-feeders have received relatively little conservation and management attention, partially due to their cryptic lifestyles and a limited appreciation of their functional significance. In this context, our findings provide valuable traction for their conservation and management, given that pelagic filtration can assist in minimising harmful effects of nutrient enrichment. Our findings additionally raise awareness for processes that restrict spatial occupancy and abundance of sandprawns to be managed appropriately to ensure that pelagic filtration and ecosystem resilience are not constrained. At a local level, prolonged mouth closure likely confines marine influence to the lower reaches in the Zandvlei Estuary, thereby imposing spatial limits on sandprawn distribution and filtration effects. Similarly, destruction of sedimentary habitats as part of coastal development, or subsequent canalisation, reduces resilience against nutrient enrichment by eliminating endobenthic filtration. In Durban Bay, on the east coast of South Africa, roughly 75% of intertidal sandflats have been lost to development between the 1890s and 1990s^56^. Such large-scale losses of sandprawn-dominated habitats are likely to have had major repercussions for water quality in the bay, based on our findings. Canalisation, which is common in estuaries locally^57^, particularly urban ones, need to be reconceptualised to be more ecologically sensitive to burrowing sandprawns and similar endobenthic fauna. As shown by our *in situ* data, canalisation can lead to increased phytoplankton biomass due to exclusion of sandprawns and their filtration capabilities. Reconfigured canal designs that permit endobenthic burrowing could allow for filtration to occur while fulfilling a stabilisation role. Lastly, unregulated harvesting of sandprawns (and similar organisms) together with trampling-induced destruction of burrows can reduce filtration effectiveness in the long-term.

Given the value of coastal ecosystems and the ever-increasing threat posed by nutrient enrichment to their socio-ecological functioning, sandprawns and functionally similar deposit-feeders may be useful additions to existing nature-based tools that can counter enrichment. This idea may be extrapolated to suspension-feeding endobenthic crustaceans, given that past work has illustrated the potential for their burrows to act as traps for suspended material^25^. Inclusion of sandprawns and similar organisms in coastal management will likely broaden the pool of functional-traits available to combat enrichment, given that they possess unique biological attributes to deal with harsh abiotic conditions, including hypoxia and hypercapnia^18,58^. Investments in appropriate management plans for these organisms and their use in bioremediation initiatives have the potential to increase coastal resilience against nutrient enrichment, and thereby enhance ecosystem functioning and the quality of goods and service provision. Overall, our work dovetails with calls that emphasise wilful ecosystem restoration initiatives and the use of nature-based solutions to counter degradation in coastal ecosystems^6,15^.
